# Utilisation pattern of ophthalmic services in Ashanti Region, Ghana

**DOI:** 10.4102/phcfm.v16i1.4326

**Published:** 2024-02-20

**Authors:** Abdul-Kabir Mohammed, Alvin J. Munsamy

**Affiliations:** 1 Department of Optometry, Faculty of Biosciences, Kwame Nkrumah University of Science and Technology, Kumas, Ghana; 2 Department of Optometry, Faculty of Health Sciences, University of KwaZulu- Natal, Durban, South Africa

**Keywords:** utilisation, ophthalmic services, visual impairment, blindness, eye care services

## Abstract

**Background:**

Best practice in optometry and ophthalmology recommends regular visits to eye care professionals, as routine eye examinations support early detection of ocular defects and associated systemic, sometimes potentially life-threatening, conditions.

**Aim:**

The study sought to determine the utilisation of ophthalmic services and its associated factors in the Ashanti region of Ghana.

**Setting:**

Fifty electoral areas in 10 of the 43 districts in the Ashanti region of Ghana.

**Methods:**

A total of 1615 participants, aged 18 years and above, were randomly selected in the Ashanti region of Ghana for this population-based, cross-sectional survey. The factors associated with having had an eye examination were guided by Andersen’s Behavioural Model. The data were analysed using multiple logistic regression, employing the IBM SPSS software, version 25.

**Results:**

After statistical adjustments, compared with the 18–29-year-old age group, older participants were found to be more likely to utilise eye care services: In addition, participants with higher formal education had higher odds for eye care utilisation compared with no former education: Being hypertensive, self-reported vision problems and feeling that regular eye examinations are important, were statistically associated with eye care utilisation.

**Conclusion:**

There is alarmingly poor utilisation of ophthalmic services in the Ashanti region of Ghana. Effective programmes to promote public health by addressing the socio-economic and individual barriers hindering the uptake of ophthalmic services in the Ashanti region of Ghana are thus necessary.

**Contribution:**

The study addresses a gap in the knowledge of factors associated with ophthalmic services utilisation in the Ashanti region of Ghana.

## Introduction

Eye care utilisation is among the essential factors considered in addressing the rising global prevalence of visual impairment and avoidable blindness.^1^ Visual impairment and blindness remain a major public health issue globally, especially in the low- and middle-income regions.^2^

Although the elimination of avoidable blindness and visual impairment, especially from infectious diseases, has seen some significant progress in developed countries, it is still a major public health issue in the developing and under-developed countries.^2^ The World Health Organization (WHO) and the International Agency for the Prevention of Blindness (IAPB), have, for more than a decade, been working towards the eradication of preventable blindness by the year 2020 through the VISION 2020: The Right to Sight initiative.^3^ Fatouhi et al.,^4^ revealed in their study that eye care service utilisation, is a crucial factor in realising the goals of ‘VISION 2020’. Therefore, understanding utilisation trends across Ghana could assist in designing strategies to improve eye care utilisation.

Best practice in optometry and ophthalmology recommends regular visits to eye care professionals, as routine eye examinations support the early detection of ocular defects and associated systemic, sometimes potentially life-threatening, conditions.^5^ Early interventions to restore, manage or treat such conditions also improve the prognosis. Furthermore, in cases of low vision where optimal sight cannot be restored, the quality of life of the patients can be improved through rehabilitation and other supportive interventions.^6^ It is essential that people access ophthalmic services regularly, because of the availability of interventions to prevent sight loss as a result of eye diseases or other causes of avoidable vision impairment.^7^ However, a study among older adults reported that the uptake of eye examinations in low, lower-middle, upper-middle and high-income countries was only 10%, 24%, 22% and 37%, respectively.^8^

Studies have been conducted on the low level of utilisation of ophthalmic services in both developed and developing countries to understand the factors responsible for the low utilisation of ophthalmic services.^9,10,11^ Studies have reported that individuals do not routinely access ophthalmic services in Ghana.^7,12,13^ However, these studies fell short of finding the reasons for the low utilisation of ophthalmic services, which is crucial in developing strategies to improve utilisation of the service.

A clear knowledge of ophthalmic service utilisation and its related factors will provide a framework, which stakeholders can use to develop strategies to improve eye care utilisation. This will reduce the prevalence of preventable blindness and visual impairment among people.

This study sought to determine the utilisation of ophthalmic services and the associated factors in the Ashanti region of Ghana, to help inform strategies to improve utilisation of the service.

## Research methods and design

### Study design

This study employed a population-based, cross-sectional descriptive design. To be included, participants had to fulfil the following requirements: residing in Ghana’s Ashanti region at the time of data collection and being at least 18 years old.

### Setting

The sample was drawn from 10 out of the 43 districts in the Ashanti region of Ghana.

### Study population and sampling strategy

The Fluid Survey Sample size calculator was used to determine a minimum sample size of 1537:

$$\text{Sample size}=\frac{\frac{z^{2}\times p(1-p)}{e^{2}}}{1+\left(\frac{z^{2}\times p(1-p)}{e^{2}N}\right)}$$ [Eqn 1]

where ***N*** = population size, ***e*** = margin of error (percentage in decimal form), ***z*** = *z*-score and ***p*** = sample proportion.

For the population of 4 780 380 in the Ashanti region of Ghana, the minimum sample size was determined to be 1537 using a 95% confidence level and a 2.5% margin of error (the desired level of precision).

A multistage sampling technique was used for this study. By means of a proportionate to population probability technique, 50 electoral areas were chosen from 10 randomly selected districts out of the 43 in the Ashanti region. Of the 10 selected districts, six (Amansie south, Atwima Nwabiagya, Bosomtwe, Kumawu, Amansie West and Offinso North) were rural while four (Asokore Mampong, Asokwa, Ejisu and Old Tafo) were urban. Upon reaching a central point in an electoral area, a bottle was spun to determine a direction. Following that 15 households were selected per electoral area along the direction, making a total of 750 households. Within the households, 1804 individuals met the inclusion criteria, out of which 1615 (89%) agreed to take part in the survey.

### Data collection

The biodata of the respondents (age, gender, education level, employment status and monthly income) as well as their eye care-seeking behaviour and utilisation were collected through a structured interviewer-guided questionnaire. Demographic information and the use of eye care services were determined with the use of items from the WHO multicountry World Health Survey questionnaire.^14^ Teaching and research assistants in the Optometry Department at Kwame Nkrumah University of Science and Technology in Kumasi, Ghana, administered all the questionnaires. The evaluation of eye care utilisation was performed by asking participants if they ever had an eye examination at an eye clinic. Andersen’s Behavioural Model^15^ was used to investigate factors associated with having had an eye examination. This model put the variables into predisposing, enabling and need categories.

Gender and age group served as study predisposing factors. The employment status, monthly income, residential location (rural or urban) and educational attainment were the enabling factors. The need factors were years since the last eye examination, having noticed a change in vision within the last 2 years and having systemic diseases (diabetes, hypertension, sickle cell anaemia). Systemic status (being afflicted by a systemic disease) was determined by self-reported history of diagnosis by a medical practitioner.

### Data analysis

The Statistical Package for the Social Sciences Software, version 25 (SPSS 25) was used to analyse the data after it was entered into a Microsoft Excel worksheet (Microsoft Inc., USA). Analysis, both descriptive and inferential, was performed. Tables were used to display the results. Regression analysis and chi-square test were used for inferential analysis. A significance level of *p* ≤ 0.05 was applied to all tests.

### Ethical considerations

Ethical approval for the study was obtained from the BREC of the University of KwaZulu-Natal, South Africa (Ref: BREC/00001787/2020) and the Committee on Human Research, Publications and Ethics of the Kwame Nkrumah University of Science and Technology, Kumasi, Ghana (Ref: CHRPE/AP/006/17). Gatekeeper consent was obtained from the Ashanti Regional Directorate of Health Services. Informed written consent was obtained from all the literate survey participants and the legally authorised representatives of illiterate participants. All study procedures adhered to the tenets of the Declaration of Helsinki.

## Results

### Sample characteristics

A total of 1615 of the 1804 individuals who were eligible participated in this study, representing a response rate of 89%. The mean age of the participants was 36.2 years, with an age range of 18–82 years. Of the participants, 54.4% were females and 52% lived in urban districts; 48.7% of the respondents were from the age group 18–29 years, followed by the 30–34-year-old group (26.7%). The majority of the respondents (87.7%) had some form of formal education and 25.6% had up to tertiary education. While 68.4% reported that they were in employment, 27.1% of the respondents earned less than 500 cedis (83 US dollars, the lowest wealth index) monthly, while only 0.4% earned more than 5000 cedis (826 US dollars, the highest wealth index). A diagnosis of one or more systemic diseases was reported by 12.3%: 3.9% were diabetic; 9.8% were hypertensive and 0.3% had sickle cell disease. Of the participants, 34.5% reported having observed a change in their vision within the last 2 years, while 85.3% of the participants reported that they felt regular eye examinations were necessary, even without symptoms, and 87.3% believed that children under five required eye examinations. All of the above-mentioned responses are shown in Table 1.

**TABLE 1:** Description of the sample.

Variable	Total (*N* = 1615)		Males (*N* = 737, 45.6%)		Females (*N* = 878, 54.4%)		χ^2^	*P*
	*n*	%	*n*	%	*n*	%		
**Predisposing factors**								
**Age groups (years)**								
18–29	787	48.7	370	50.2	417	47.5	7.922	0.094
30–44	431	26.7	204	27.7	227	25.9	-	-
45–59	227	14.1	102	13.8	125	14.2	-	-
60–74	127	7.9	47	6.4	80	9.1	-	-
≥ 75	43	2.7	14	1.9	29	3.3	-	-
**Enabling factors**								
**District**								
Amansie South	129	8.0	53	7.2	76	8.7	9.148	**< 0.001**
Asokore Mampong	279	17.3	134	18.2	145	16.5	-	-
Asokwa	215	13.3	97	13.2	118	13.4	-	-
Atwima Nwabiagya	182	11.3	89	12.1	93	10.6	-	-
Bosomtwe	186	11.5	62	8.4	124	14.1	-	-
Ejisu	151	9.3	75	10.2	76	8.7	-	-
Kumawu	101	6.3	34	4.6	67	7.6	-	-
Amansie West	116	7.2	41	5.6	75	8.5	-	-
Offinso North	60	3.7	27	3.7	33	3.8	-	-
Old Tafo	196	12.1	125	17.0	71	8.1	-	-
**Level of education**								
None	199	12.3	62	8.4	137	15.6	10.254	**< 0.001**
Primary	124	7.7	29	3.9	95	10.8	-	-
Intermediate	449	27.8	172	23.3	227	31.5	-	-
Secondary	429	26.6	215	29.2	214	24.4	-	-
Tertiary	414	25.6	259	35.1	155	17.7	-	-
**Employed**	1103	68.3	527	71.5	576	65.6	6.447	**0.011**
**Monthly income (cedis)**								
≤ 500	438	27.1	164	22.3	274	31.2	5.766	**< 0.001**
501–1000	364	22.5	182	24.7	182	20.7	-	-
1001–2000	220	13.6	117	15.9	103	11.7	-	-
2001–4999	75	4.6	59	8.0	16	1.8	-	-
≥ 5000	6	0.4	5	0.7	1	0.1	-	-
**Need factors**								
**Presence of systemic disease**								
Diabetes	63	3.9	17	2.3	46	5.2	0.482	0.488
Hypertension	159	9.8	52	7.1	107	12.2	2.204	0.138
Sickle cell disease	4	0.3	0	0.0	4	0.5	2.230	0.135
Observed change in vision in the last 2 years	557	34.5	228	30.9	329	37.5	7.557	**0.006**
Think children under five need eye examinations	1410	87.3	639	86.7	771	87.8	0.446	0.504
Think regular eye examinations are important	1377	85.3	655	88.9	722	82.2	14.546	**< 0.001**
Visit eye clinic any time they have an eye problem	279	17.3	126	17.1	153	17.4	0.030	0.984
**Period since last eye examination**								
Less than a year	211	13.0	87	11.8	124	14.1	2.745	**< 0.001**
1 to 2 years	194	12.0	110	14.9	84	9.6	-	-
2 to 3 years	96	5.9	57	7.7	39	4.4	-	-
3 to 5 years	85	5.3	33	4.5	52	5.9	-	-
More than 5 years	82	5.1	39	5.3	43	4.9	-	-
Don’t remember	23	1.4	7	0.9	16	1.8	-	-

Note: The significant *p*-values are the ones boldfaced.

*N*, total sample per category; *n*, frequency; %, percentage frequency; χ^2^, Pearson chi-square value.

### Eye care utilisation among the respondents

Table 2 shows the percentage of participants who had ever had an eye examination and whether they visit an eye clinic whenever they have an eye problem, across selected variables. The result in Table 2 was analysed by means of chi-square test. Overall, only 42.8% of the participants had ever had an eye examination and 17.3% always visit an eye facility whenever an eye problem arose.

**TABLE 2:** Eye care utilisation and related factors among the participants.

Variable	Ever had an eye examination				Visit eye clinic whenever one has an eye problem				*n* = 1615
	(*n* = 691)	% (42.8)	χ^2^	P*	(*N* = 279)	% (17.30)	χ^2^	P*	
**Predisposing factors**									
**Gender**									
Males	333	45.2	3.181	0.073	126	17.10	0.031	0.984	737
Females	358	40.8	-	-	153	17.40	-	-	878
**Age (Years)**									
18–29	285	36.2	10.117	**< 0.001**	124	15.80	10.546	**< 0.001**	787
30–44	166	38.5	-	-	58	13.50	-	-	431
45–59	122	53.7	-	-	59	26.00	-	-	227
60–74	80	63.0	-	-	30	23.60	-	-	127
≥ 75	38	88.4	-	-	8	18.60	-	-	43
**Enabling factors**									
**District**									
Amansie South	40	31.0	6.177	**< 0.001**	17	13.20	6.595	**< 0.001**	129
Asokore Mampong	144	51.6	-	-	55	19.70	-	-	279
Asokwa	86	40.0	-	-	27	12.60	-	-	215
Atwima Nwabiagya	67	36.8	-	-	23	12.60	-	-	182
Bosomtwe	109	58.6	-	-	64	34.40	-	-	186
Ejisu	71	47.0	-	-	25	16.60	-	-	151
Kumawu	23	22.8	-	-	4	4.00	-	-	101
Amansie West	39	33.6	-	-	8	6.90	-	-	116
Offinso North	17	28.3	-	-	5	8.30	-	-	60
Old Tafo	95	48.5	-	-	51	26.00	-	-	196
**Level of education**									
None	86	43.2	4.595	**< 0.001**	28	14.10	3.914	**< 0.001**	199
Primary	38	30.6	-	-	20	16.10	-	-	124
Intermediate	146	32.5	-	-	45	10.00	-	-	449
Secondary	173	40.3	-	-	47	11.00	-	-	429
Tertiary	248	59.9	-	-	139	33.60	-	-	414
Employment status
Employed	463	41.9	1.264	0.334	191	17.30	0.413	0.531	1103
Unemployed	228	44.6	-	-	88	17.20	-	-	512
**Monthly income (Cedis)**									
≤ 500	119	27.1	9.592	**< 0.001**	48	11.00	**9.884**	< 0.001	438
5001–1000	143	39.3	-	-	52	14.30	-	-	364
1001–2000	140	63.6	-	-	66	30.00	-	-	220
2001–4999	56	74.7	-	-	24	32.00	-	-	75
≥ 5000	5	83.3	-	-	1	16.70	-	-	6
**Need factors**									
Diabetes	37	58.7	8.384	**0.004**	6	9.50	9.172	**0.002**	63
Hypertension	124	78.0	13.374	**< 0.001**	42	26.40	6.251	**0.012**	158
Sickle cell disease	2	40.0	2.655	0.103	2	40.00	0.871	0.351	5
Observed change in vision in the last 2 years	338	60.7	12.325	**< 0.001**	148	26.60	6.132	**< 0.001**	557
Thinks regular eye examinations are important	612	44.4	10.989	**0.002**	236	17.10	0.150	0.985	1377
Thinks children under five need eye examinations	617	43.8	4.291	**0.038**	248	17.60	1.099	0.577	1410

Note: The significant *p*-values are the ones boldfaced.

*N*, total sample per category; *n*, number of responses; %, percentage of responses; χ^2^, Pearson Chi-Square value.

#### Predisposing factors

A higher percentage of males had had an eye examination, compared with females (45.2% vs. 40.8%). However, this was not statistically significant. Utilisation of eye care services increases with age, as 88.4% of participants in the age group ≥ 75 years had had an eye examination, compared with 36.2% in the 18–29-year age group. Table 2 also shows that age was significantly associated with visiting an eye care facility whenever an eye problem arose (*p* < 0.001).

#### Enabling factors

Bosomtwe and Asokore Mampong were the only 2 of the 10 districts where more than 50% of the participants (58.6% and 51.6%, respectively) had ever had an eye examination. Kumawu, which is one of the rural districts, registered the lowest (22.8%) eye care utilisation. While 59.9% of participants who were educated up to the tertiary level had sought eye care, all other education levels reported less than 50% utilisation of eye care services.

The study also found increased eye care utilisation as income rose. Whereas 83.3% of participants who earn ≥ 5000 cedis had had an eye examination, only 27.1% of those earning ≤ 500 cedis had had an eye examination. Although the employed appeared to have utilised eye care services less than the unemployed (41.9% vs. 44.6%), this was not statistically significant (*p* = 0.334). Among the enabling factors, constituency, level of education and monthly income were significantly (*p* < 0.001) associated with visiting an eye clinic whenever an eye problem arose.

#### Needs factors

Whereas 78.0% of hypertensives and 58.8% of diabetics had sought an eye examination, only 40% of participants with sickle cell anaemia had ever utilised eye care. Hypertension and diabetes were found to be significantly associated with accessing eye care (hypertension, *p* < 0.001; diabetes, *p* = 0.004). Of the participants who self-reported vision problem in the last 2 years, 60.7% had ever had an eye examination, compared with 44.4% of those who felt regular eye examinations were necessary. However, both groups were significantly associated with seeking eye care (*p* < 0.001 and *p* = 0.002, respectively). With regard to need factors, diabetes (*p* = 0.004), hypertension (*p* < 0.001) and vision problems (*p* < 0.001) were significantly associated with accessing eye care whenever an eye problem arose.

### Factors associated with reports of a previous eye examination

Table 3 shows the results of the multiple logistic regression models on whether the participants had ever had an eye examination and the associated factors.

**TABLE 3:** Multiple logistic regression: Having had a previous eye examination and related factors.

	Model 1			Model 2			Model 3		
	OR	95% CI	*P*	OR	95% CI (OR)	*P*	OR	95% CI (OR)	*P* *
**Predisposing factors**									
**Gender**									
Males	Ref	-	-	Ref	-	-	Ref	-	-
Females	0.77	0.63–0.95	**0.014**	1.09	0.86–1.37	0.484	0.95	0.75–1.21	0.665
**Age (Years)**									
18–29	Ref	-	-	Ref	-	-	Ref	-	-
30–44	1.10	0.87–1.41	0.429	1.12	0.84–1.49	0.455	1.73	0.04–0.32	**-0.001**
45–59	2.06	1.53–2.78	**-0.001**	2.52	1.73–3.68	**-0.001**	2.12	0.04–0.36	**-0.001**
60–74	3.09	2.09–4.56	**-0.001**	4.96	3.15–7.80	**-0.001**	3.17	0.05–0.47	**0.001**
≥ 75	14.00	5.44–36.02	**-0.001**	28.98	10.60–79.19	**-0.001**	12.24	0.08–0.69	**0.008**
**Enabling factors**									
**District**									
Amansie South	-	-	-	Ref	-	-	Ref	-	-
Asokore Mampong	-	-	-	2.00	1.23–3.27	**0.006**	0.64	0.37–1.11	0.111
Asokwa	-	-	-	1.36	0.82–2.26	0.239	1.36	0.88–2.08	0.166
Atwima Nwabiagya	-	-	-	1.17	0.69–1.98	0.567	0.83	0.52–1.31	0.422
Bosomtwe	-	-	-	3.44	2.05–5.80	**-0.001**	0.76	0.48–1.22	0.258
Ejisu	-	-	-	2.14	1.25–3.66	**0.006**	2.51	1.54–4.07	**-0.001**
Kumawu	-	-	-	0.82	0.43–1.58	0.552	1.44	0.88–2.33	0.144
Amansie West	-	-	-	1.29	0.71–2.35	0.408	0.05	0.28–1.01	0.052
Offinso North	-	-	-	0.79	0.37–1.69	0.541	0.34	0.43–1.34	0.343
Old Tafo	-	-	-	1.39	0.82–2.35	0.221	0.07	0.23–1.05	0.067
**Level of education**									
None	-	-	-	Ref	-	-	Ref	-	-
Primary	-	-	-	0.73	0.43–1.25	0.246	1.31	0.19–0.51	**-0.001**
Intermediate	-	-	-	1.01	0.67–1.53	0.964	1.15	0.08–0.26	**-0.001**
Secondary	-	-	-	1.96	1.27–3.01	**0.002**	2.25	0.17–0.35	**-0.001**
Tertiary	-	-	-	3.48	2.18–5.56	**-0.001**	3.51	0.36–0.71	**-0.001**
**Employment status**									
Unemployed	-	-	-	Ref	-	-	Ref	-	-
Employed	-	-	-	0.81	0.59–1.11	0.196	0.42	0.04–4.16	0.456
**Monthly income (Cedis)**									
≤ 500	-	-	-	Ref	-	-	Ref	-	-
5001–1000	-	-	-	1.67	1.21–2.31	**0.002**	0.39	0.04–3.90	0.421
1001–2000	-	-	-	2.91	1.98–4.29	**-0.001**	0.60	0.06–6.03	0.668
2001–4999	-	-	-	3.84	2.08–7.10	**-0.001**	1.09	0.11–10.87	0.944
≥ 5000	-	-	-	3.77	0.40–35.84	0.248	1.25	0.12–12.99	0.854
**Need factors**									
Diabetes	-	-	-	-	-	-	1.82	0.92–3.62	0.088
Hypertension	-	-	-	-	-	-	1.30	0.18–0.50	**-0.001**
Sickle cell disease	-	-	-	-	-	-	2.48	0.35–17.76	0.367
Observed change in vision in the last 2 years	-	-	-	-	-	-	2.31	0.26–0.45	**-0.001**
Think regular eye exam is important	-	-	-	-	-	-	2.19	0.41–0.87	**0.007**
Think children under 5 years need eye examination	-	-	-	-	-	-	1.14	0.78–1.68	0.494

Note: The significant *p*-values are the ones boldfaced.

Model 1 assessed predisposing variables in a univariate model; Model 2 considered predisposing and enabling variables; Model 3 included predisposing, enabling and need variables.

OR, odds ratio; CI, confidence interval; Ref, reference group;

* , Pearson chi-square.

#### Model 1: Predisposing factors

People in the older age group showed an increased likelihood of having previously accessed eye care, compared with those between 18 and 29 years of age: 45–59 years, OR = 2.06, *p* < 0.001; 60–74 years, OR = 3.09, *p* < 001; ≥ 75 years, OR = 14.0, *p* < 001. Also, when compared with males, females had a reduced likelihood of previously seeking eye care (OR = 0.77; *p* = 0.014).

#### Model 2: Predisposing and enabling factors

In Model 2, which included predisposing and enabling factors, the associations with predisposing factors were similar to those in Model 1. Regarding the enabling factors, Model 2 showed that participants in Asokore Mampong, Bosomtwi and Ejisu had an increased likelihood of utilising eye care compared with those in Amansie South (Asokore Mampong, OR = 2.00, *p* = 0.006; Bosomtwi, OR = 3.44, *p* < 0.001; Ejisu, OR = 2.14, *p* = 0.006). Model 2 also showed that having higher education was significantly associated with an increased likelihood of having previously accessed eye care when compared with those who did not have any form of formal education (secondary, OR = 1.96, *p* = 0.002; tertiary, OR = 3.48, *p* < 0.001). Model 2 went on to show that increased wealth was associated with an increased likelihood of previously accessing eye care, when compared with the lowest wealth index (5001 – 1000, OR = 1.67, *p* = 0.002; 1001 – 2000, OR = 2.91, *p* < 0.001, 2001 – 5000, OR = 3.84, *p* < 0.001).

#### Model 3: Predisposing, enabling and need factors

The final model, Model 3, incorporated the predisposing, enabling and need factors. After statistical adjustment, the older age groups were found to be significantly associated with seeking eye care, compared with the 18–29-year-old age group: 30–44 years, OR = 1.73, *p* < 0.001; 45–59 years, OR = 2.12, *p* < 0.001; 60–74 years, OR = 3.17, *p* = 0.001; ≥ 75 years, OR = 12.24, *p* = 0.008. With regard to districts, Model 3 showed that only participants in Asokore Mampong had an increased likelihood of accessing eye care when compared with Amansie South (Asokore Mampong, OR = 2.51, *p* < 0.001). Model 3 also showed that education, when compared with no formal education, was significantly associated with eye care utilisation: primary, OR = 1.31, *p* < 0.001; intermediate, OR = 1.15, *p* < 0.001; secondary, OR = 2.25, *p* < 0.001; tertiary, OR = 3.51, *p* < 0.001. Model 3 further showed that being hypertensive (OR = 1.30, *p* < 0.001), having self-reported vision problems in the last 2 years (OR = 2.31, *p* < 0.001) and feeling that regular eye examinations are important (OR = 2.19, *p* = 0.007), were statistically associated with eye care utilisation.

## Discussion

Coverage is a crucial component in the assessment of healthcare system performance. In this study, therefore, participants’ self-reported history of ever having visited an eye care facility for their eye care needs was used as a measure of eye care utilisation. This study revealed that 57.2% of the participants had never had their eyes examined, while only 25% had had their eyes examined at least once in the previous 2 years. The study found that age, district of residence, level of education, monthly income, presence of systemic disease (diabetes, hypertension), self-reported vision problems and feeling that regular eye examinations are important, were significantly associated with eye care utilisation. Gender was not statistically associated with eye care utilisation in this study. However, a higher proportion of males had utilised eye care, compared with females.

More than half (57.2%) of the participants had never utilised eye care services, which was in agreement with several other studies.^7,16,17,18,19^ Possible factors that could serve as barriers to ophthalmic services utilisation include availability, affordability and accessibility.

This study found age to be significantly associated with eye care utilisation. The increased likelihood of the aged seeking eye care, compared with the young, can be attributable to age-related ophthalmic conditions.^4^ The older one gets, the higher one’s risk of developing ophthalmic conditions and the more likely one is to seek eye care.^3^ This is consistent with studies in both developing^18^ and developed^20^ countries.

District of residence was also found to be associated with eye care utilisation. Residents in Asokore Mampong, Ejisu and Bosomtwi were significantly more likely to seek eye care than those in other districts. Regional differences in eye care utilisation have been reported by other studies.^18,21^ This may be attributed to differences in awareness about the need for regular eye examinations and the availability of ophthalmic services in the communities. Asokore Mampong and Ejisu are urban districts that have a number of public and private eye care facilities available to their people. Although Bosomtwi is a rural constituency, it has two well-equipped ophthalmic mission clinics operated under the auspices of the Christian Health Association of Ghana (CHAG), which serve patients across the region. This may be one of the reasons why a higher proportion of the participants in Bosomtwi, compared with the other districts, were associated with seeking eye care, despite it being a rural district.

Level of education was associated with eye care utilisation. Participants with a higher level of education were more likely to utilise eye care than the less educated. This may be attributed to greater knowledge and perhaps more concern about their eye health. Fotouhi et al.^4^ also reported an increased likelihood of utilising eye care with higher levels of education in an Iranian population and presumed educated people usually belong to the higher socioeconomic class, which facilitated greater access to eye care in terms of affordability. Other studies^13,22,23^ also reported a positive association between education and eye care use.

Monthly income was also found to be associated with increased likelihood of utilising eye care services. A plausible explanation could be the general socioeconomic status of higher-income earners. They may, therefore, find ophthalmic services more affordable and thus have greater access compared with low-income earners. This observation was reported in other low- and middle-income countries.^11,24,25,26^

Self-reported vision problems were also associated with an increased likelihood of utilising eye care services, as such problems may affect the activities of daily living. Vision can also be a determinant in the career aspirations of people, and thus its loss is linked to the loss of certain occupations, which may drive poverty and mental health conditions, among others. Ahmad et al.^27^ and Akuffo et al.^25^ reported a higher probability of persons with vision-related problems seeking eye care services. People with symptoms of blurred vision, or any other vision-related problem, will generally be more likely to seek eye care.

Participants who felt regular eye examinations were important were significantly associated with an increased likelihood of seeking eye care. People who are aware of the need for regular eye examinations will know more about the preventative aspects of vision loss than those who are unaware, thus, increasing their likelihood of using eye care services. Regular eye examinations can detect ophthalmic conditions in their earliest stage when they are most treatable to prevent vision loss, as many vision-threatening eye diseases including glaucoma, macular degeneration, cataracts or diabetic retinopathy have no or minimal symptoms until the disease has progressed.

A significant association was also found between the presence of a systemic disease, such as diabetes or hypertension and eye care utilisation. This could be a result of the recommendation generally given to diabetics and hypertensives to monitor their eyes at least once every 2 years^28^ because of the risk of developing retinopathies after some years of having these conditions. Other studies^29,30^ reported similar observations. They recommended health education and awareness campaigns on the benefits of seeking ophthalmic services timeously to prevent visual impairment.

To the authors’ knowledge, this is a pioneering study assessing ophthalmic service utilisation in the Ashanti region of Ghana, with the major advantage of using population-based evidence for eye care utilisation. This suggests that its findings could be generalised to the entire population of the Ashanti region of Ghana. However, our study has some limitations. The factor used to measure the utilisation of ophthalmic services was self-reported, which may have been affected by recall bias: recalling the period since the last eye examination. Data were generated from 10 of the 43 districts in the region because of logistical constraints. Nonetheless, the results from this study could assist in developing strategies to improve ophthalmic service utilisation in the Ashanti region and in Ghana as a whole.

## Conclusion

In conclusion, the study revealed an alarmingly low use of ophthalmic services in the Ashanti region of Ghana. Ophthalmic service utilisation was found to be associated with some predisposing, enabling and need factors, as explained by Anderson’s healthcare utilisation model. Regarding predisposing factors, ophthalmic service utilisation was associated with age but not with gender. Enabling factors such as district of residence, level of education and monthly income (wealth) were associated with an increased likelihood of seeking ophthalmic services. With regard to the need factors, we found that participants with diabetes, hypertension, vision problems and those who think regular eye examinations are important are associated with eye care utilisation.

There is a need for effective public health programmes to address the socio-economic and individual barriers hindering the uptake of ophthalmic services in the Ashanti region of Ghana. The importance of timeously seeking eye care services in preventing visual impairments and blindness needs to be emphasised. The awareness of available eye care services in the region, with an emphasis on the implications of delays in seeking eye care, should be promoted intensively. Stakeholders should start public eye health campaigns on the annual, dedicated World Sight Day to raise awareness about the need for regular eye examinations.
